# Evolution of HIV virulence in response to widespread scale up of antiretroviral therapy: a modeling study

**DOI:** 10.1093/ve/vew028

**Published:** 2016-10-03

**Authors:** Joshua T. Herbeck, John E. Mittler, Geoffrey S. Gottlieb, Steven M. Goodreau, James T. Murphy, Anne Cori, Michael Pickles, Christophe Fraser

**Affiliations:** 1 International Clinical Research Center, Department of Global Health, University of Washington, Seattle, WA 98104, USA; 2 Department of Microbiology, University of Washington, Seattle, WA 98195, USA; 3 Departments of Medicine, University of Washington, Seattle, WA 98195, USA; 4 Department of Anthropology, University of Washington, Seattle, WA 98195, USA; 5 Department of Infectious Disease Epidemiology, Imperial College London, London W2 1PG, UK

**Keywords:** HIV, virulence, antiretroviral therapy, evolution, model, viral load

## Abstract

There are global increases in the use of HIV antiretroviral therapy (ART), guided by clinical benefits of early ART initiation and the efficacy of treatment as prevention of transmission. Separately, it has been shown theoretically and empirically that HIV virulence can evolve over time; observed virulence levels may reflect an adaptive balance between infected lifespan and per-contact transmission rate. However, the potential effects of widespread ART usage on HIV virulence are unknown. To predict these effects, we used an agent-based stochastic model to simulate evolutionary trends in HIV virulence, using set point viral load as a proxy for virulence. We calibrated our model to prevalence and incidence trends of South Africa. We explored two distinct ART scenarios: (1) ART initiation based on HIV-infected individuals reaching a CD4 count threshold; and (2) ART initiation based on individual time elapsed since HIV infection (a scenario that mimics “universal testing and treatment” (UTT) aspirations). In each case, we considered a range in population uptake of ART. We found that HIV virulence is generally unchanged in scenarios of CD4-based initiation. However, with ART initiation based on time since infection, virulence can increase moderately within several years of ART rollout, under high coverage levels and early treatment initiation (albeit within the context of epidemics that are rapidly decreasing in size). Sensitivity analyses suggested the impact of ART on virulence is relatively insensitive to model calibration. Our modeling study suggests that increasing HIV virulence driven by UTT is likely not a major public health concern, but should be monitored in sentinel surveillance, in a manner similar to transmitted resistance to antiretroviral drugs.

## 1. Introduction

Worldwide, 15 million HIV-infected individuals are receiving antiretroviral therapy (ART) (UNAIDS 2014). To take advantage of the benefits of earlier treatment initiation for both individual (better disease prognosis—Lundgren et al. 2015) and population endpoints (decreased rates of onward transmission—Cohen et al. 2011), UNAIDS has set ambitious 90–90–90 targets for 2020 (90% of HIV-infected individuals will know their HIV status; 90% of individuals with diagnosed HIV infection will receive ART; 90% of individuals receiving ART will have viral suppression). Considerable attention has been devoted to studies of implementation, costs, emergence of drug resistance, and population-level effectiveness. However, whether ART may affect the evolution of HIV has received scant attention. If theoretical models suggest that widespread ART use could result in the emergence of more (or less) virulent HIV infections, this should motivate efforts to monitor virulence evolution.

HIV virulence, defined here as the rate of disease progression in untreated infections, is commonly estimated via the proxies of set point viral load (SPVL; the viral load after the resolution of acute infection but prior to AIDS), baseline CD4+ T-cell count (the first CD4 count after the resolution of acute infection), rate of CD4+ T-cell decline, level of immune activation, or viral replicative capacity. Modeling studies of HIV virulence have suggested that, in epidemics where ART use is not widespread, HIV may adaptively evolve toward an intermediate level of virulence, to balance the per-contact transmission rate with the infected lifespan (Fraser et al. 2007; Shirreff et al. 2011; Herbeck et al. 2014). These findings are consistent with the “trade-off” theory of virulence evolution, where an optimal virulence should exist that maximizes the (pathogen’s) lifetime transmission success (Anderson and May 1982; Ewald 1983). For example, low virulence (low SPVL) will result in decreased infectivity but more lifetime transmissions in untreated infections (due to longer infected lifespans); high virulence (high SPVL) will result in higher infectivity but fewer total transmissions (due to shorter infected lifespans) (Fraser et al. 2007).

HIV virulence models have focused on SPVL, as SPVL is prognostic for the rate of disease progression (Mellors et al. 1995, 1997, 2007; de Wolf et al. 1997). Importantly, SPVL is a viral phenotype with the necessary requirements for adaptive evolution: variation in the population (Korenromp et al. 2009; Herbeck et al. 2012); correlation with the per-contact transmission rate (fitness) (Quinn et al. 2000; Fideli et al. 2001; Gray et al. 2001; Pilcher et al. 2004; Baeten et al. 2011); and at least partially determined by genotype (i.e. heritable across transmission pairs) (Alizon et al. 2010; Hecht et al. 2010; Hollingsworth et al. 2010; van der Kuyl et al. 2010; Fraser et al. 2014).

Large-scale “treatment as prevention” or “universal test and treat” programs will likely shift the distribution of HIV transmissions by individual stage of infection (Eaton and Hallett 2014), potentially modifying the balance between per-contact transmission rate and the length of the infected lifespan. It is possible that biomedical interventions that extend the lifespans of HIV-infected individuals will shorten the (effective) viral lifespans and result in increased HIV virulence: less virulent viruses will no longer reap the benefits of longer infected lifespans (we define an “effective viral lifespan” as the time of infection until ART is initiated, after which there are large decreases in viral load and transmission potential, assuming high adherence to ART). Alternatively, it is possible that individuals infected with the most virulent viruses will initiate treatment the earliest, thus providing an added evolutionary advantage to less virulent viruses. In short, viruses that transmit the most before treatment initiation may have a selective advantage. However, because of the range of possible interactions and counter-acting selection pressures, these evolutionary scenarios are hard to intuit, and so need to be studied within the context of a mathematical model. It is important to identify specific ART-related scenarios in which HIV virulence could evolve, and how rapidly virulence could change.

Our goal was to predict, using a stochastic, agent-based model, the effect of ART scale-up on HIV virulence. We used SPVL as a virulence proxy to investigate whether ART can apply evolutionary pressure on the balance between HIV per-contact infectivity and the infected lifespan. In addition, we tested whether different ART scenarios of eligibility and initiation, in the form of historical (based on CD4 counts) or anticipated (“universal test and treat”) treatment guidelines, mediate this pressure. Our model allowed for fine-scale variation in virulence (individual SPVL), and allowed for population-based levels of virulence (mean SPVL) to change over time.

## 2. Materials and Methods

We previously developed a stochastic, agent-based HIV evolutionary and epidemic model that simulates viral dynamics within and between individuals (Herbeck et al. 2014). This model allows for an HIV virulence phenotype (SPVL) to change over the course of a simulated epidemic, and thus provides an evolutionary framework in which a balance can be achieved between infectivity (efficiency of viral transmission) and virulence (rate of disease progression). The underlying model was written in C with a front-end written in R. The code is freely available from the authors, upon request.

### 2.1. Simulated population

Each epidemic simulation starts with *N* total individuals at time 0 (HIV-uninfected and infected), with each infected individual (of *n* total HIV-infected individuals) provided an SPVL value randomly selected from a normal distribution with user-defined mean and variance (Table 1). Entry of new (HIV-uninfected) individuals into the population occurs at a constant rate, such that the overall population will stay at its initial value. All individuals are equally resistant and/or susceptible to infection, with respect to the per-contact transmission rate. 

**Table 1.** Parameters of the model and initial values.

Parameter	Value
Demographic and behavioral	
Initial overall population size	1 × 10^5^ individuals
Initial number of infected	2 × 10^3^ individuals
Minimum relationship duration	0.1 years
Maximum relationship duration	5.0 years
Subgroups defined by relationship duration*	Original: <0.5, 0.5–2.5, >2.5 years
	Alternate: 1 group (no subgroups)
Probability of sexual contact, in each group*	Original: 1.0, 0.05, 0.03 per day
	Alternate: 1.0
Mean degree*	Original: 0.9
	Alternate: 0.7
Virologic	
Viral load at time zero	10 copies/ml
Viral load at peak viremia	1.0 × 10^7^ copies/ml (Schacker et al. 1998; Pilcher et al. 2004)
Time to peak viremia	21 days (Schacker et al. 1998; Pilcher et al. 2004)
Total time of acute infection	91 days (Pilcher et al. 2004)
Viral load progression rate, natural log	0.05 per year (Geskus et al. 2007)
Viral load at AIDS (CD4<200)	5.0 × 10^6^ copies/ml (Geskus et al. 2007)
SPVL	
Variance of log_10_ SPVL	0.7 (Korenromp et al. 2009)
Heritability of SPVL across transmissions (h^2^)	0.36 (Fraser et al. 2014)
Mutational variance	0.2
Transmission	
Maximum transmission rate	0.005/day (Fraser et al. 2007; Shirreff et al. 2011)
Viral load at 0.5 max transmission rate	13,938 copies/ml (Fraser et al. 2007; Shirreff et al. 2011)
Hill coefficient, transmission function	1.02 (Fraser et al. 2007; Shirreff et al. 2011)
Shape parameter, transmission function	3.46 (Fraser et al. 2007; Shirreff et al. 2011)
Disease progression	
See Cori *et al.* for CD4 wait time matrix stratified by SPVL (Cori et al. 2015)	
ART	
Time to initiation after becoming eligible by CD4	1 year(Eaton et al. 2012)
Viral load after ART initiation	50 copies/ml

Parameters with an asterisk (*) had different values between the original and alternate model calibrations.

### 2.2. Viral load parameters

The model includes the following parameters related to HIV viral load: (1) the distribution of SPVL in a population of HIV-infected individuals (Korenromp et al. 2009; Herbeck et al. 2012); (2) the daily progression of viral load over the course of an individual infection, including distinct viral load trajectories for acute, chronic, and AIDS stages (Schacker et al. 1998; Bonhoeffer et al. 2003; Pilcher et al. 2004; Geskus et al. 2007); (3) the predictive relationship between HIV viral load and the per-day transmission rate (assuming a given probability of sexual contact per day; see below) (Fraser et al. 2007; Shirreff et al. 2011); (4) the predictive relationship between SPVL and the rate of disease progression, mediated through rates of individual CD4+ T cell decline that are stratified by individual SPVL (Cori et al. 2015); and (5) a viral role in the determination of each individual’s SPVL (i.e. variation in the viral genotype explains a portion of the population variation in SPVL; non-zero heritability of SPVL in the infected population) (Alizon et al. 2010; Hecht et al. 2010; Hollingsworth et al. 2010; van der Kuyl et al. 2010; Fraser et al. 2014). Parameter estimates for these viral components were fixed based on relevant literature (Table 1), and parameter uncertainty was addressed systematically by sensitivity analyses reported in our previous description of the model (Herbeck et al. 2014), and by performing our experiments on two separate model calibrations. See Supporting information for further descriptions of the above model functions.

### 2.3. Primary model calibration

We performed a two-step process to calibrate our primary model, with the intent to reproduce incidence and prevalence trajectories based on prevalence data from South Africa (Health SADo 2011), so that the output of our main analysis was directly comparable to the twelve HIV epidemic models described in Eaton et al. (2012). As such, we first used evidence-based (i.e. viral load, CD4) parameter values within our evolutionary model; these are discussed in depth in our previous description of the model, and in the Supporting information (Herbeck et al. 2014). Second, we calibrated assumption-based (i.e. behavioral) parameter values specifically to produce epidemic trends similar to those observed in South Africa, and based on similar calibration of the twelve models included in Eaton et al. (2012).

It is not straightforward to calibrate HIV epidemic models to accurately reflect the decreases in incidence and prevalence observed in epidemics of sub-Saharan Africa (Nagelkerke et al. 2014); as in all epidemic models, it is widely accepted that the observed epidemic trends in sub-Saharan Africa require relatively complex assumptions about population structure (different risk groups), patterns of sexual contact, or changes in risk behavior over time (Nagelkerke et al. 2014; Funk et al. 2015). The twelve models in Eaton et al. all dealt with this issue, and used varying degrees of assumption-based parameter values in their calibration; none were able to reproduce realistic epidemic dynamics without some underlying epidemiological complexity (Eaton et al. 2012). Our choice of behavioral parameters to include in this primary model, based on an epidemic with a core group of individuals with increased transmission rates, and the parameter settings of which produced the calibrated model output, follow from previous HIV epidemic models. This approach allowed us to externally validate our model epidemic output, and to interpret potential clinical and epidemiological impacts of our evolutionary output in a realistic (and accepted) framework. Further explanation of our model parameterization and calibration is included in the Supporting information.

### 2.4. Alternate model calibration

It was not the goal of our overall study to assess the potential effects of variation in behavioral parameters on the interaction of ART and HIV virulence evolution. Rather, our goal was to assess the effects of ART on HIV virulence evolution, with ART applied under a wide variation of scenarios and coverage, while maintaining the primary epidemic calibration based on South Africa and the twelve models in Eaton et al. However, we assessed whether the predicted effects of ART on virulence evolution were robust to variation in the overall epidemic scenarios (different variants of the underlying epidemic model, including underlying parameterization and resulting incidence and prevalence trends). To do this, we modified our behavioral parameters by: (1) decreasing the mean degree (the average number of sexual contacts each person has at a given time); (2) removing the core group of individuals that had short relative relationship durations and elevated rates of sexual contact; (3) incorporating a random mixing sexual network that eliminated the assortative mixing of sexual contacts by relationship duration category (Table 1). With this alternate epidemic model we repeated the entirety of our ART-based experiments.

### 2.5. Antiretroviral therapy parameters

We structured ART dynamics as a heuristic starting point for theoretical studies of ART and HIV virulence evolution. As such, we assumed a single, standard regimen, with complete adherence and retention, and without the emergence of drug resistant mutations and associated changes in viral fitness (numerous studies report that transmitted drug resistance is rare in most populations (Alizon et al. 2009; Roberts, Goulder, and McLean 2015) and that the vast majority of transmitted drug resistance mutations have low fitness costs (Gandon and Day 2009; Bolker, Nanda, and Shah 2010)). Individual ART use was applied after fulfilling necessary criteria of *eligibility threshold, initiation time*, and *population coverage*. We evaluated two types of ART eligibility scenarios. First, eligibility was based on CD4 count thresholds (entry into a CD4 count category: CD4 >500; CD4 <500; CD4 <350; CD4 <200)). These scenarios are most relevant to how treatment has been implemented in recent years, as countries have followed WHO guidelines. Second, eligibility was based on time elapsed since date of infection (1, 2, 3, 4, 5, or 6 years after infection). These scenarios reflect likely changes in the future as treatment becomes nearly universally available through the UNAIDS 90–90–90 initiative.

Initiation of ART after an individual became eligible was immediate for the “time since infection” eligibility criterion but was delayed for 1 year for the “CD4 count category” criterion (this delay in ART initiation for those reaching a CD4 eligibility threshold was consistent with the models compared in Eaton et al. (2012), and realistic given testing rates in South Africa). No individuals were eligible for ART during acute infection in any scenario (regardless of CD4 count category). Population-level ART coverage was implemented via individual probabilities of initiating ART, after meeting the eligibility criteria described above. Our model includes complete adherence to ART. As such, our ART population coverage parameter settings are likely overestimates of coverage in populations with less than complete adherence (as fewer individuals will be on ART if adherence is not 100%). Decreases in transmission probability for individuals receiving ART were mediated entirely by an immediate decrease in viral load to 50 copies/ml upon treatment initiation.

### 2.6. Simulations and output

We ran epidemic simulations for 60 years, in discrete time-steps of 1 day. For each model run we tracked the distribution (mean, median, and variance) of SPVL (viral load at the end of acute infection and prior to initiation of ART) and the population incidence and prevalence. For specific comparisons to the models described in Eaton et al. (2012), we evaluated changes to mean SPVL, incidence, and person-years of ART per infection averted that were observed at 8 and 38 years after the roll-out of a population-level ART program that began in 2012—with treatment eligibility at CD4 count <350 cells/μl and population coverage at 80% (compared with counterfactuals in the same populations without ART). This specific treatment scenario was used by Eaton et al. (2012) to approximate an implementation of World Health Organization guidelines (current at the time of that study) and the Joint United Nations Programme on HIV/AIDS definition of “universal access” as reaching 80% of HIV-infected individuals.

Thus, we began our simulations in year 1990, ART was introduced to the populations in the beginning of year 2012, and evaluations were done using output from the midpoint of years 2020 (8 years) and 2050 (38 years). In addition to the specific comparison to Eaton et al. outputs, we evaluated combinations of ART coverage (40–100%, by 20% increments) and either ART time since infection eligibility (1, 2, 3, 4, 5, or 6 years after infection) or ART CD4 count threshold eligibility (all eligible (CD4 >500), CD4 <500, CD4 <350, or CD4 <200). For each combination of coverage and eligibility we performed ten replicate simulations.

## 3. Results

### 3.1. Calibration

**Figure 1.**
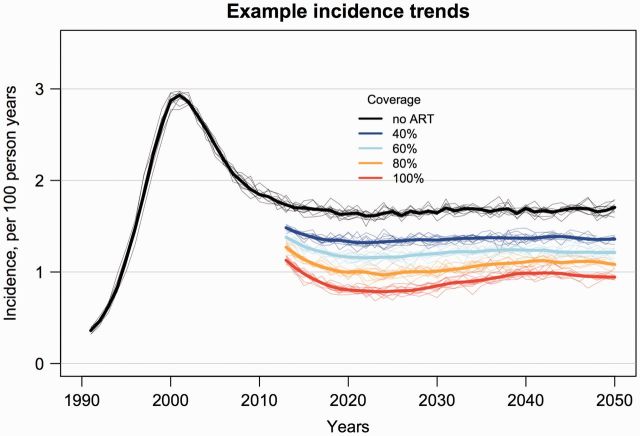
Simulated trends in HIV incidence for ART scenarios of 40, 60, 80, and 100 percent coverage (individual probability of receiving ART with complete adherence) and CD4 count threshold for treatment initiation <350 cells/ml, versus the counterfactual epidemic simulation with no ART. Shown are LOESS regression lines for ten random replicates for each ART coverage scenario (thin lines), and the mean of these replicates (thick lines). Initial mean SPVL was 4.5 log_10_ copies/ml.

Our primary results focus on the calibration of a stochastic, agent-based model (Herbeck et al. 2014) to the HIV epidemic of South Africa (Table 1). We chose to calibrate the model in this way first, because the epidemic in South Africa is one of the largest, globally, and second, to make our study explicitly and readily comparable to the twelve models of ART and HIV incidence that were documented and compared by Eaton et al. (2012). These twelve models, and comparisons among them represent the fullest attempt, to date, at quantifying the potential impact of ART on HIV incidence in a high-incidence generalized epidemic. These twelve models included simulations starting in 1990 with ART rollout starting in 2012. The models were compared based on epidemiological endpoints measured at 8 and 38 years after ART rollout (2020 and 2050, respectively); we followed this same structure. Our simulations were initiated with 2% HIV prevalence and initial mean SPVL of 3.5, 4.5, or 5.5 log_10_ copies/ml. Incidence (per 100 person years) rose quickly to ∼3%, followed by a decline to ∼1.75% and stabilization around year 2010 (Fig. 1). Prevalence rose to ∼12%, followed by a decline to ∼8%. 20 percent of transmissions occurred from individuals in their first year of infection; the majority of transmissions occurred in chronic infection. These epidemic outputs, in the absence of ART, were similar to outputs from the twelve models compared by Eaton et al. (2012). 

### 3.2. Virulence evolution in the absence of ART

Starting with initial SPVL distributions that reflected low, intermediate, and high virulence (mean SPVLs of 3.5, 4.5, and 5.5 log_10_ copies/ml, respectively), HIV evolved toward an intermediate level of virulence (Supplementary Fig. S1). In addition, as predicted under stabilizing selection, the population variance of SPVL decreased over time in all scenarios. Under the current model assumptions, the evolutionary optimal mean SPVL, in the absence of ART, is predicted to be ∼4.70 log_10_ RNA copies/ml. We have previously performed sensitivity analyses for the effects of viral and behavioral parameters on SPVL levels and SPVL evolution in our model (Herbeck et al. 2014); we summarize these here. The inferred optimal virulence (mean SPVL) was insensitive to variation in the following viral parameters: (1) rate of viral load increase in chronic infection (*s* from Equation 1 in Supplementary information); (b) maximum transmission rate (*B*_max_ in Equation 2 in Supplementary information); (c) maximum time to AIDS; and (d) peak viremia in acute infection. The rate at which mean SPVL evolved to an inferred population-level optimum (Supplementary Fig. S1) depended principally on *B*_max_; mean SPVL increased more rapidly in the early years of epidemics with higher *B*_max_ values, before arriving at similar mean SPVL levels (Herbeck et al. 2014). The inferred optimal virulence (mean SPVL) was sensitive to the mean degree of the sexual network, defined as the average number of sexual partners that each person has at any given time. Higher mean degrees were associated with higher mean SPVL. Because we were interested in comparing mean SPVL between simulations with and without ART, this sensitivity did not affect our main findings. However, our alternate model calibration, performed as a sensitivity analysis and described below, includes a lower mean degree and thus lower mean SPVL.

### 3.3. Virulence evolution in the presence of ART

#### 3.3.1. Time since infection thresholds for ART initiation, representing universal test and treat

**Figure 2.**
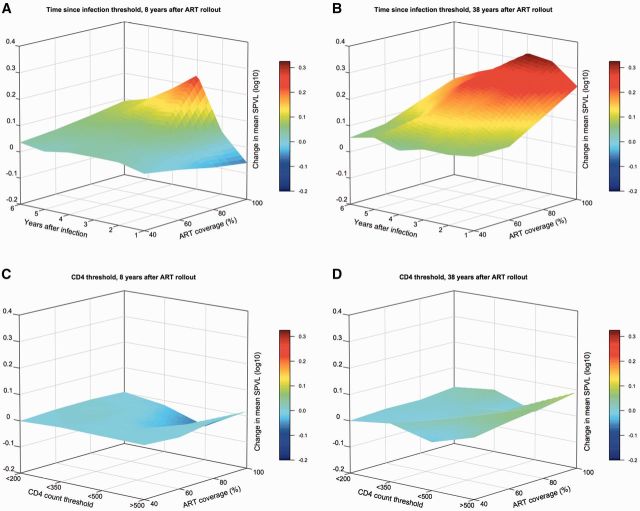
Surface plots showing change in mean SPVL between epidemic simulations with and without ART, for scenarios of increasing ART coverage (individual treatment probability) and ART initiation based either on time since infection or CD4 count threshold. For epidemic scenarios with ART initiation based on time since infection, (**A**) shows mean SPVL change 8 years after ART rollout (from year 2012 to 2020), and (**B**) shows mean SPVL change 38 years after rollout (from year 2012 to 2050). For epidemic scenarios with ART initiation based on CD4 count, (**C**) and (**D**) show mean SPVL at 8 and 38 years after rollout, respectively. Linear interpolation was used to estimate mean SPVL values for scenarios in which epidemics were extinguished prior to measurement (see Supplementary Figs. S2 and S5).

**Figure 3.**
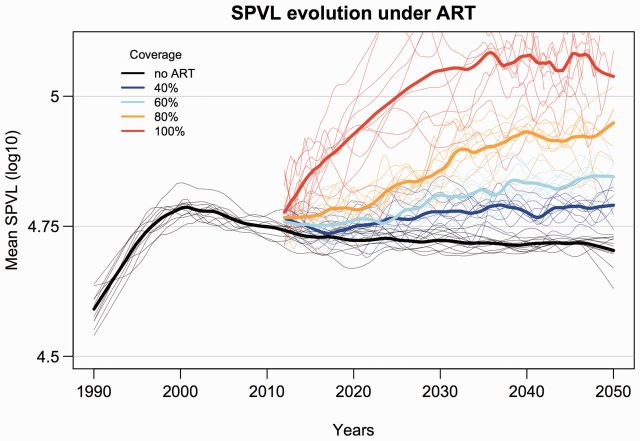
Example simulated trends in HIV virulence (mean SPVL) for scenarios of 40, 60, 80, and 100 percent coverage (individual probability of treatment) and ART initiation at 3 years elapsed after infection, versus the counterfactual simulation with no ART. Shown are LOESS regression lines for ten random replicates for each ART coverage scenario (thin lines), and the mean of these replicates (thick lines). Initial mean SPVL was 4.5 log_10_ copies/ml. ART coverage lines start at year 22, corresponding to a simulation starting at year 1990 with ART rollout at year 2012.

With ART initiation thresholds based on time elapsed since infection (without consideration of CD4 count or stage of infection), virulence was slightly increased by 2020 (8 years after ART rollout), except in scenarios with 100% coverage, in which mean SPVL was increased by 0.2 log_10_ with initiation at 3 years after infection (Fig. 2). By 2050, however, increased virulence was seen at all coverage levels, at all initiation times. (We used linear interpolation to estimate the mean SPVL values for simulations in which epidemics were ended prior to measurement at 8 or 38 years after rollout—see Supplementary Figs. S2 and S5.) The largest increases were seen with ART initiation at 2, 3, and 4 years after infection and ART coverage at and above 60%. In these scenarios the maximum increases in mean SPVL were ∼0.4 log_10_ copies/ml by 2050 (e.g. from ∼4.7 to ∼5.1 log_10_ copies/ml) (Fig. 2 and Supplementary Fig. S2). These increases in virulence only occurred in the context of very large reductions in incidence (Supplementary Fig. S3). Figure 3 shows the specific example of increasing mean SPVL for ART initiation at 3 years after infection, for ART coverage ranging from 40 to 100 percent. Similar plots for SPVL trends under each of the evaluated time thresholds, at increasing coverage levels, are shown in the Supporting information (Supplementary Fig. S4). 

#### 3.3.2. CD4 count thresholds for ART initiation

With CD4 count thresholds for ART initiation, mean SPVLs at 8 and 38 years after ART rollout (2020 and 2050) were generally unchanged between ART and counterfactual (no ART) simulations (Fig. 2 and Supplementary Fig. S5). Small decreases in virulence (decreases in mean SPVL up to ∼0.10 log_10_ copies/ml) were seen by 2020 for the CD4 <500 ART initiation threshold at higher coverage levels (80 and 100 percent), with the magnitude of change greater with increasing coverage. These decreases in virulence were not maintained by 2050. Slight increases in virulence were seen by 2050 in the CD4 >500 scenario (all individuals are eligible for ART) with high coverage; incidence was near zero in these scenarios (Supplementary Fig. S6). Additional plots for SPVL trends under the examined range of CD4 thresholds, at increasing coverage levels, are shown in the Supporting Information (Supplementary Fig. S7).

### 3.4. Incidence trends in the presence of ART

Our model produced estimates of person-years of ART needed per infection averted, and reductions in incidence, that were similar to estimates described in Eaton et al. (2012) (Table 2). In ART simulations with 80% coverage and an ART initiation threshold of CD4 <350, we observed a mean percent reduction in incidence of 40.03 percent (SD 2.31) by 2020 (8 years after ART rollout) and of 36.82 percent (SD 2.92) by 2050. In these simulations, the mean person-years of ART needed per infection averted was 7.42 (SD 0.22) and 8.60 (SD 2.47) in 2020 and 2050, respectively. As expected, we observed greater declines in incidence as ART coverage or CD4 count eligibility thresholds increased (Supplementary Fig. S6). Reassuringly, incidence declined even in the ART scenarios that led to increasing HIV virulence. 

**Table 2.** Simulated impacts of ART, comparing models with evolving HIV virulence (a distribution of individual SPVL values and allowing for evolutionary change) and static SPVL (all individuals have SPVL of 4.5 log_10_ copies/ml and individual SPVLs are identical across transmissions).

	Time since infection threshold		CD4 count threshold	
	Static SPVL	Evolving SPVL	Static SPVL	Evolving SPVL
Percent reduction in incidence				
Year 2020	53.99 ±2.51	54.27 ±2.92	32.42 ±2.83	40.03 ±2.31
Year 2050	43.61 ±4.03	38.72 ±2.23	31.42 ±2.45	36.82 ±3.46
Person-years of ART per infection averted				
Year 2020	7.52 ±0.24	6.08 ±0.22	9.98 ±0.19	7.42 ±0.22
Year 2050	9.39 ±1.67	7.59 ±0.46	12.80 ±1.75	8.60 ±2.47

Two ART scenarios are shown for evolving and static SPVL models: ART initiation and eligibility at CD4 <350 cells/μl and 80 percent coverage; and ART initiation and eligibility at 4 years elapsed after infection and 80 percent coverage. Values are means and standard deviations for ten replicate model runs.

### 3.5. Incidence trends for evolving versus static SPVL

Our HIV epidemic model includes a distribution of virulence levels among individuals and allows virulence (mean SPVL) to evolve. To assess the potential effects of these model characteristics on standard epidemic output, we repeated our simulations with a single, time invariant SPVL (4.5 log_10_ copies/ml) and 100 percent heritability (i.e. all individuals have SPVL = 4.5 over the course of a simulation), and compared the output of this static SPVL model with the output of the evolving SPVL model.

With a variable and evolving SPVL, the predicted benefits of ART are greater than predicted by a model with static SPVL (in an epidemic scenario of 80 percent ART coverage and an ART initiation threshold of CD4 <350) (Table 2). With evolving SPVL, incidence reductions are greater by years 2020 and 2050 (e.g. ∼40 versus ∼32 percent reduction by year 2020), and fewer person-years of ART are required per infection averted (e.g. 7.42 person-years versus 9.98 person-years at year 2020; 8.60 versus 12.80 person-years by year 2050).

### 3.6. Results from the alternate model calibration

We repeated our simulation experiments with alternate parameterization of the epidemic model, as a test of the sensitivity of our primary results (above) to the overall model calibration. These alternate epidemic simulations were initiated with 2 percent HIV prevalence and initial mean SPVL of 4.5 log_10_ copies/ml; incidence (per 100 person-years) rises at a slower rate than in our primary model, but continues rising to ∼5% incidence by the end of the epidemic simulation (Supplementary Fig. S8). This alternate model produced results, with respect to HIV virulence evolution, equivalent to those obtained from the primary model calibration: ART initiation based on CD4 count results in generally unchanged HIV virulence (mean SPVL relative to the no ART counterfactual), while ART initiation based on time since infections results in moderate increases in mean SPVL, in scenarios of early initiation and high coverage (Supplementary Figs. S9, S10 (time trends), and S11 (CD4 trends)).

## 4. Discussion

HIV virulence evolution has attracted a considerable amount of recent interest (Fraser et al. 2007; Herbeck et al. 2012). Separately, the effects of ART on HIV transmission, incidence, and prevalence are issues of critical public health importance (Cohen et al. 2011; Eaton et al. 2012). In our analysis we have used a mathematical model to jointly examine these inter-related aspects of HIV evolution and epidemiology.

### 4.1. ART scenarios and virulence evolution

Based on our model, we predict that widespread ART use will, overall, have minimal effects on HIV virulence. However, specific scenarios may yield clinically significant increases in SPVL: when ART initiation is based solely on the time elapsed since infection (rather than a CD4 count threshold), when ART initiation occurs relatively early after infection, and when ART population coverage is relatively high (at or above 60%). The maximum predicted increases in mean SPVL were ∼0.4 log_10_ copies/ml by 2050, 38 years after ART rollout (e.g. from ∼4.7 to ∼5.1 log_10_ copies/ml). In these scenarios, the beneficial impact of ART programs could be partially mitigated by the emergence of highly virulent viruses; nonetheless, the model predicts substantial and durable reductions in incidence for these scenarios.

Table 3 compares clinically relevant results (i.e. proxies for disease progression) of the two distinct ART scenarios, including (1) initiation at CD4 <350 and 80 percent coverage (the ART scenario for the primary model comparisons described in Eaton et al. 2012), and (2) initiation at 3 years after infection (also with 80 percent coverage). In the latter time since infection scenario, which represents “universal test and treat”, our model predicts increases in mean SPVL of 0.27 log_10_ copies/ml by 2050 (4.72–4.97) (Fig. 3). Following the transmission and disease progression functions of our model (see Materials and Methods and Supporting Information), an increase of this magnitude results in a ∼8% increase in annual transmission rate (infectivity) and a ∼13% decrease in time to CD4 <350. This change in infectivity hinges on the shape of our viral load and transmission function (Equation 2 in Supporting information), which assumes that infectivity plateaus at high viral loads (Fraser et al. 2007). If, rather, infectivity does not plateau but rather continues to increase with higher viral loads, increases in transmission rate due to virulence evolution will be greater (Lingappa et al. 2010; Hughes et al. 2012); previous studies (using different underlying functions) have predicted that SPVL increases of 0.50 log_10_ will decrease the median time to AIDS by 3 years (Gupta et al. 2007), and will increase the annual transmission rate by up to 37 percent (Quinn et al. 2000; Fideli et al. 2001; Fraser et al. 2007; Lingappa et al. 2010). 

**Table 3.** Impact of HIV virulence evolution given in terms of: mean SPVL; infectiousness (mean annual transmission rate); and years until specific CD4+ T-cell counts.

	Mean SPVL	Mean annual transmission rate	Mean years	Mean years	Mean years
			To CD4 <500	To CD4 <350	To CD4 <200
No ART	4.72	0.76	2.4	4.47	7.03
A. With ART; CD4<350	4.7	0.75	2.3	4.27	6.86
B. With ART; 3 years after infection	4.97	0.82	2.05	3.91	6.25
C. With ART; 4 years after infection	4.9	0.81	2.18	4.14	6.73

The baseline comparison for simulated epidemics without ART is shown. ART scenarios with 80 percent coverage are shown for three eligibility types: (A) ART eligibility at CD4 <350 cells/μl; (B) ART eligibility at 3 years elapsed after infection; and (C) ART eligibility at 4 years elapsed after infection. Values are means for all new infections between 2045 and 2050 (33–38 years after ART, the last 5 years of 60-year epidemic runs), for ten replicates each, for the SPVL (viral load at the end of primary infection).

The predicted dynamics of virulence evolution (Fig. 2) suggest that the selective advantage of high virulence is greatest with early ART initiation times after infection (<4 years after infection). That is, if both low and high virulence viruses have the same infection duration (due to treatment), then high virulence viruses will have an advantage due to their higher transmission rates. As the time threshold for ART initiation increases (up to 6 years and beyond), the observed change in mean SPVL (HIV virulence) decreases—because these scenarios are approximating the natural history of HIV without ART, and the evolutionary balance between high virulence (short lifespans with high infectivity) and low virulence (long lifespans with low infectivity) is unaffected by ART.

The generally stable virulence level predicted by the ART scenarios with CD4 count thresholds is perhaps surprising, given that the mean times to these CD4 thresholds are similar to the time thresholds for initiation that produced increased virulence (i.e. 3–5 years after infection). This can be explained by the selective advantage of higher virulence viruses being mitigated by the selective earlier removal of these same high virulence lineages (by ART), relative to lower virulence lineages. In effect, ART initiation based on CD4 count appears to balance the selective processes for and against more virulent viruses, while ART initiation based on time elapsed since infection provides a selective advantage *only* for more virulent viruses. This can be seen in the relative proportions of transmissions that occur after ART rollout, for high (defined here as >4.70 log_10_ copies/ml, the observed population average, 4.7) and low (<4.70 log_10_ copies/ml) virulence viruses (Supplementary Fig. S12).

We note that ART initiation (and alteration of the effective viral lifespan) may be analogous to parasite life history events. The potential impact of the timing of parasite life history events on virulence evolution has been described previously (Day and Burns 2003); in which Day notes that discounting the effects of future events (transmissions that occur later in infections) may lead to increased virulence. It is this type of scenario, in our simulations, that leads to increased virulence: when ART is initiated relatively early after infection, the selective advantage of lower virulence (in terms of transmissions accumulated after more virulent viruses have killed their hosts) is lost.

### 4.2. Can ART explain empirical (observed) virulence trends?

The fastest mean linear increase in SPVL (estimated after ART rollout, from 2012 to 2050) was 0.008 log_10_ copies/ml/year, for 100% coverage and ART initiation at 3 years after infection (Fig. 3); 50 percent slower than the SPVL increase observed in the largest cohort study completed with date (0.016 log_10_ copies/ml/year; 95% CI 0.013–0.019) (Pantazis 2014), and 40 percent slower than the summary trend from a meta-analysis of eight published SPVL trends (0.013 log_10_ copies/ml/year; 95% CI −0.001 to 0.03) (Herbeck et al. 2012). Given that our maximum rate was produced in a scenario with 100 percent coverage, we infer that the empirical SPVL trends are likely not due to ART rollout alone—although we cannot absolutely rule out that differences in the primary transmission routes of our modeled epidemic (heterosexual sex) and the empirical estimates (mostly men-who-have-sex-with-men epidemics in North America and Europe) would result in different effects of ART on virulence.

It has been suggested that decreases in HIV virulence (measured by the proxies of viral replicative capacity and viral load) observed in ART-naïve antenatal cohorts in Gaborone, Botswana (compared with Durban, South Africa) may be due partially to historical increases in ART coverage (and more substantially to the extent of viral adaptation to the host immune response) (Payne et al. 2014). Our model does not simulate the evolution of viral replicative capacity *per se*, but we can assess whether the observed difference in median viral loads (4.19 and 4.47 log_10_ copies/ml, respectively, in Gaborone and Durban) can be explained by ART. A decline of this magnitude (∼0.3 log_10_ copies/ml; *ceteris paribus*) in median VL is not seen in our model when comparing ART and counterfactual simulations; the largest decreases due to ART (median decrease of ∼0.1 log_10_ copies/ml) occurred either: (A) 38 years after ART rollout, or (B) at high CD4 threshold (<500) and 100 percent coverage within 8 years of rollout—two scenarios not consistent with the history of ART in Botswana. A recent meta-analysis estimated the mean CD4 count at ART initiation in southern Africa to be 152 cells/μl (Siedner et al. 2015). Thus, the majority of HIV infected individuals are likely starting ART late in infection, regardless of a given CD4-based guideline. Our model predicts that this will result in moderate increases in HIV virulence, not decreased virulence. Qualitatively, then, we infer that ART has not contributed to the postulated decreased HIV virulence in Botswana.

## 5. Conclusions

Our evolutionary and epidemiological model predicts that under certain ART scenarios, which align closely with the “treatment as prevention,” “universal test and treat,” and “90–90–90” scenarios that HIV public health programs aspire to implement, HIV virulence may increase relatively rapidly. (At the time of writing, South Africa has implemented 2015 WHO treatment guidelines, and ART initiation will no longer be based on CD4 count as of September 2016.) These results are seen both in a model calibrated based on South Africa HIV incidence and prevalence trends and in an alternate model with entirely different parameterization. We note that incidence declines in these scenarios of increased virulence, but individuals with untreated infections will progress more quickly and per-act transmission rates will rise. These results are consistent with theoretical explorations of the effects of treatment on pathogen virulence, a key observation of which was that increasing treatment rates resulted in increasing optimal virulence (Porco et al. 2005); we observed this same result, as increasing ART coverage resulted in increasing virulence. Our results, with respect to CD4 thresholds for ART initiation, are also qualitatively consistent with a recent study that used a deterministic model to predict the effects of ART on the relative frequencies of two HIV strains representing high or low virulence (Roberts, Goulder, and McLean 2015). Further study is warranted to assess these HIV-specific predictions, including modeling aspects of combination prevention programs that may modulate these effects, for example, PrEP, medical male circumcision, or condom use, and using cohort data to evaluate empirical relationships between trends in ART coverage and HIV virulence markers.

An additional conclusion from our study is that standard HIV epidemic models (models that do not include parameters related to population-level variation in SPVL and to the capacity for HIV virulence to evolve) may underestimate the benefits of ART prevention programs. It may be beneficial, as HIV epidemiologic models continue to develop, to include realistic functions of viral evolutionary dynamics in such models. Even in worst case scenarios, our modeling study suggests that increasing virulence driven by universal test and treat is likely not a major public health concern, with a risk far outweighed by the benefits of improved clinical outcomes and reduced incidence. Changing virulence is amenable to being monitored alongside transmitted drug resistance in sentinel surveillance.

This question was recently addressed by Roberts, Goulder, and McLean (2015), who concluded on the basis of their modeling study that increasing ART use was unlikely to have a major impact on virulence. Roberts *et al.* considered the competition between two strains of differing virulence, and modeled the transmission dynamics using deterministic ordinary differential equations (Roberts, Goulder, and McLean 2015). Our study addressed a number of issues not included in the model of Roberts, Goulder, and McLean (2015), which earlier theoretical studies have all indicated may affect predictions on the evolution of virulence. First, quantitative details on the trade-off between infectiousness and rate of disease progression are important (Fraser et al. 2007; Alizon et al. 2009), and our model incorporates both the parameterization and functional forms derived from primary data. Second, epidemic dynamics have been shown to feed back into virulence trends (Gandon and Day 2009; Bolker, Nanda, and Shah 2010), and so we calibrated our model to the epidemic dynamics of South Africa. Third, demographic stochasticity may affect virulence (Griette, Raoul, and Gandon 2015), so our model is an individual-based stochastic model. Finally, contact network structure may affect virulence evolution (van Baalen 2002; Danon et al. 2013), so our model accounts for the dynamics of sexual partnership formation and dissolution. So, while our findings are broadly similar to those reported by Roberts, Goulder and McLean (2015) , this study considers a much wider range of factors that could affect virulence, and explicitly contrasts distinct scenarios of ART rollout and eligibility. Similarity of findings increases confidence in the generality of these findings.

## Supplementary data

Supplementary is available here.

## Funding

This study was supported by grants from the U.S. National Institutes of Health (R01AI108490 to J.T.H., J.E.M., and S.G., NIAID cooperative agreement UM1AI068619 to A.C., M.P. and C.F, and P30AI027757 to the University of Washington Center for AIDS Research). The content is solely the responsibility of the authors and does not necessarily represent the official views of the National Institutes of Health. C.F. is also supported by European Research Council AdG PBDR-268540 “BEEHIVE”. The funding sources had no role in the writing of this manuscript or the decision to submit it for publication.

Conflict of interest: None declared. 

## Supplementary Material

Supplementary Data
