# Supplementary material for: Evolution of HIV virulence in response to widespread scale up of antiretroviral therapy: a modeling study
Source: Virus Evol. 2016 Oct 3;2(2):vew028. doi: 10.1093/ve/vew028 (PMC5822883; doi:10.1093/ve/vew028)

Evolution of HIV virulence in response to widespread scale up of antiretroviral therapy: a modeling study

Joshua T. Herbeck[1]*, John E. Mittler[2], Geoffrey S. Gottlieb[1,3], Steven Goodreau[4], James T. Murphy[4], Anne Cori[5], Michael Pickles[5], and Christophe Fraser[5]

[1]Department of Global Health, University of Washington, Seattle, WA 98104
[2]Department of Microbiology, University of Washington, Seattle, WA 98195
[3]Departments of Medicine, University of Washington, Seattle, WA 98195
[4]Department of Anthropology, University of Washington, Seattle, WA 98195
[5]Department of Infectious Disease Epidemiology, Imperial College London, London W2 1PG, UK

*Correspondence to: herbeck@uw.edu, +1-206-520-3826.

SUPPLEMENTARY MATERIAL

Additional details of the epidemic model

Within-host component (viral load dynamics)

For each individual, upon HIV-1 infection with an initial viral population of size V_0, viral load increases exponentially at rate r until a user-defined peak viremia, V_{peak}, at a user-defined time t_{peak}. Viral load then declines exponentially at an individual-specific decay rate, d_{acute}, until it reaches set point viral load (SPVL), V_{sp}, at time t_{sp}. We define t_{sp}, the time when V_{sp} is established, in the number of days after infection; this is a user-defined length from initial infection through peak viremia until set point is reached. After reaching set point (t_{sp}), the viral load increases as (Equation 1):

$$V(t) = \mathrm{V}_{sp}\mathrm{e}^{s(t-tsp)} \quad (1)$$

where s is the user-defined parameter of annual viral load increase rate, and t is the time that has elapsed since the patient was first infected. For all HIV-1 infected individuals, we assume a log linear change in viral load after SPVL has been established until the onset of AIDS. Functionally this can be set to zero change, equivalent to stable viral load in the asymptomatic stage. Viral load upon onset of AIDS is defined as the same for all individuals, and is independent of SPVL. To reconcile population variation in SPVL (V_{sp}) with primary infection dynamics, we assume that V_0, r, t_{peak}, t_{sp} and s are the same in all individuals, but that $d_{acute} = ln(V_{peak}/V_{sp})/(t_{sp} - t_{peak})$ varies.

Within-host parameters for disease progression (CD4+ T-cell count parameters)

To model the relationship between SPVL and the rate of disease progression, as well as incorporate ART initiation thresholds based on CD4+ T cell counts, we included an intermediary function that related individual SPVL to the starting CD4 count category (individual CD4 count immediately after infection), and to subsequent disease progression based on waiting times in four CD4 count categories: CD4>500; 500>CD4>350; 350>CD4>200; CD4<200 (AIDS). This function is based on data from the AIDS Therapy Evaluation in the Netherlands (ATHENA) observational cohort, which includes HIV-infected individuals followed in the 27 HIV treatment centers in the Netherlands since 1996 [38].

Across-host component (viral load and transmission)

We assume that transmission rates follow available data from serodiscordant heterosexual partners; the probability of a HIV-infected person will transmit to a HIV-negative person is determined by an increasing Hill function (Equation 2) that follows Fraser [4]:

$$B = B_{\max} \frac{V^a}{V^a + K^a} \qquad (2)$$

where B_{max} is the maximum probability of transmission per year, V is the donor's viral load at the time of sexual contact, a is a Hill Coefficient that influences the steepness of the response curve, and K is the viral load at which the probability of transmission to a susceptible person is $B_{max}/2$. We have followed the parameter values from Fraser [4], except for B_{max}, which we have increased (from 0.001 per day in Fraser [4]) to account for the slower epidemic growth rates than expected, which may be explained as this value being from serodiscordant couples that are enrolled in HIV-1 cohorts and likely an underestimate relative to the general population.

Across-host component (heritability of set point viral load)

The SPVL, V_{sp}, is determined by both viral (the viral genotype, *VirCont*) and environmental (a combination of undefined host and environmental factors, *EnvCont*) factors. *VirCont* is different for each individual; HIV-infected individuals inherit the value of *VirCont* from the individual who infected them, with random variance introduced within a separate mutational variance parameter. Mathematically, we assume, for donor (i) and recipient (j) (Equations 3 - 5):

$$VirCont_{[j]} = VirCont_{[i]} + norm\_rand(0, MutationalVariance) \qquad (3)$$

$$EnvCont_{[j]} = norm\_rand(0, sqrt((1 - h^2) * VarianceLogSP0)) \qquad (4)$$

$$\log[V_{sp}]_{[j]} = VirCont_{[j]} + EnvCont_{[j]} \qquad (5)$$

where h = the square root of h^2, the user-defined heritability.

Sexual mixing network

For each individual in a simulation, the model maintains a list of sexual partnerships and viral transmission pairs. Individuals were not marked by sex, and risks of infection were bidirectional, meaning that both partners have independent probabilities of infecting the other partner that are dependent on individual viral loads.

For any one simulation, prior to the simulation of viral transmission, an initial set of contacts is formed by randomly choosing pairs from the population. The probability of each person entering into this link is set to e^{-L_i}, where L_i is the number contacts that person i has (for this analysis we did not allow for concurrent relationships). If this probability was not met (or if L_i > *MaxLinks*), another partner is selected at random from the population. This process is repeated until the total number of links equals $N*M/2$, where N is the total number of sexual active individuals, and M is the mean degree. The probability of a connection between individuals i and j dissolving is set to 2/(*Duration*[*i*] * *Duration*[*j*]), where *Duration*[*x*] is the expected time that person x stays in a relationship. After removing links from all newly dissolved partnerships, $N*M/2$ - $L/2$ links are added to the system, where N is the number of individuals after accounting for births and deaths that

occurred that day and *L* is the number of links in the system after the dissolution step. If this quantity is negative, no links are added.

Behavioral parameters

The virologic and CD4-based progression functions were embedded in a host population without explicit demographic (sex, age) heterogeneity, but with behavioral heterogeneity. Each individual was assigned a relational duration propensity (range: 6-60 months); when two individuals partner, their relationship was slated to last for the mean of these individual effects. The daily probability of sex varied by relationship duration (100% for relationships <= 6 months; 5% for relationships >6 months and <=30 months; 3% for relationships >30 months). The coital frequencies for the two higher categories were selected as part of the process of calibrating the model to epidemic data. Individuals were placed into relationship partnerships randomly, with no concurrency (those already in a relationship were not eligible to form a new partnership). The rate of partnership formation was a calibrated parameter value. We did not include a change in behavior over time, *e.g.* a reduction in the sexual contact rate over calendar time.

Supplementary Material Figure S1. Simulated trends in HIV virulence (via set point viral load (SPVL) as a proxy) reveal adaptive evolution toward an optimum SPVL. Shown are LOESS regression lines for ten random replicates for each initial mean SPVL (thin lines), and the mean of these replicates (thick lines).

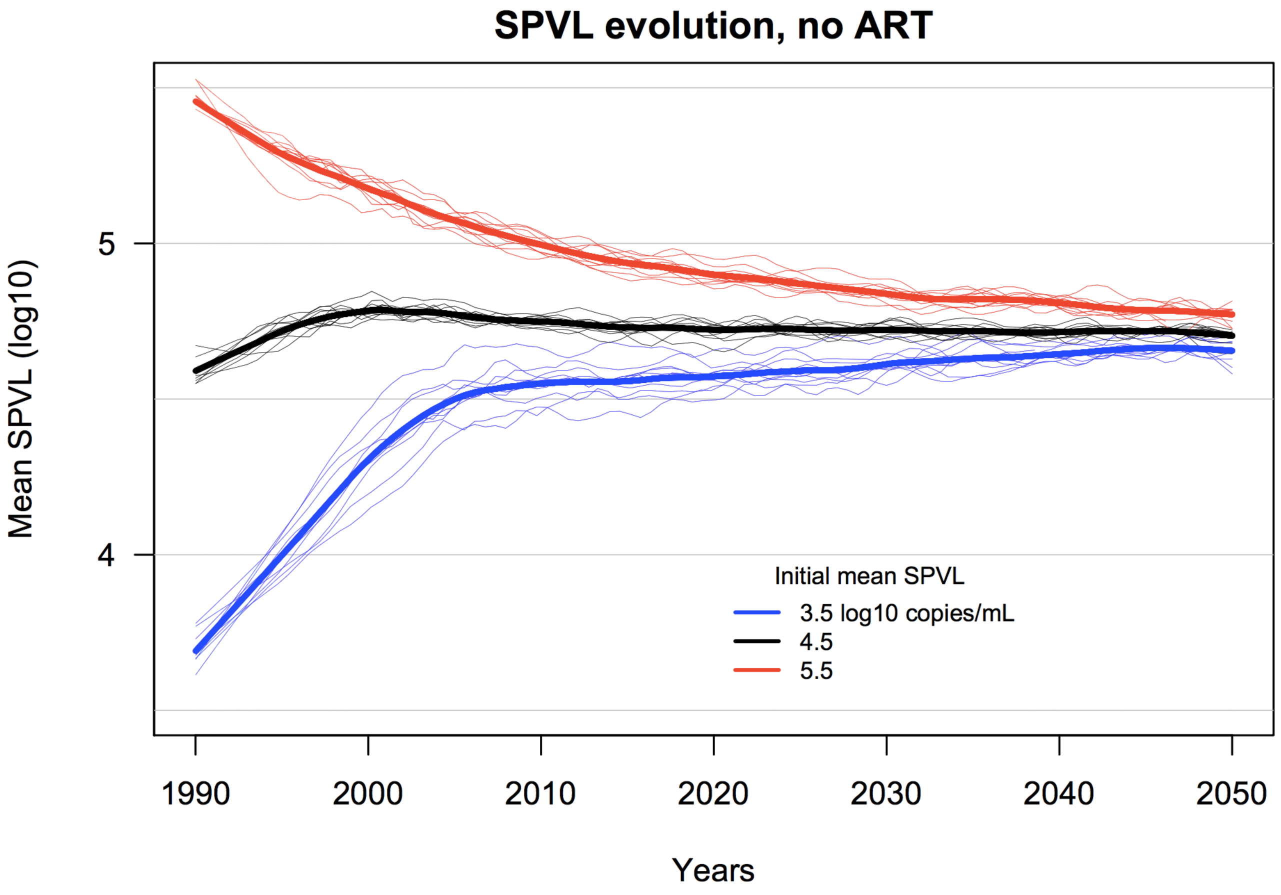

Supplementary Material Figure S2. Simulated effects of ART on HIV virulence evolution, measured at **A)** 8 and **B)** 38 years after rollout of ART (*i.e.* from ART rollout in 2012 to 2020 and 2050). ART initiation is determined based on time elapsed since infection. (Increases in mean SPVL for 100% coverage are not reported for initiation at one and two years after infection because incidence is 0 in those scenarios.)

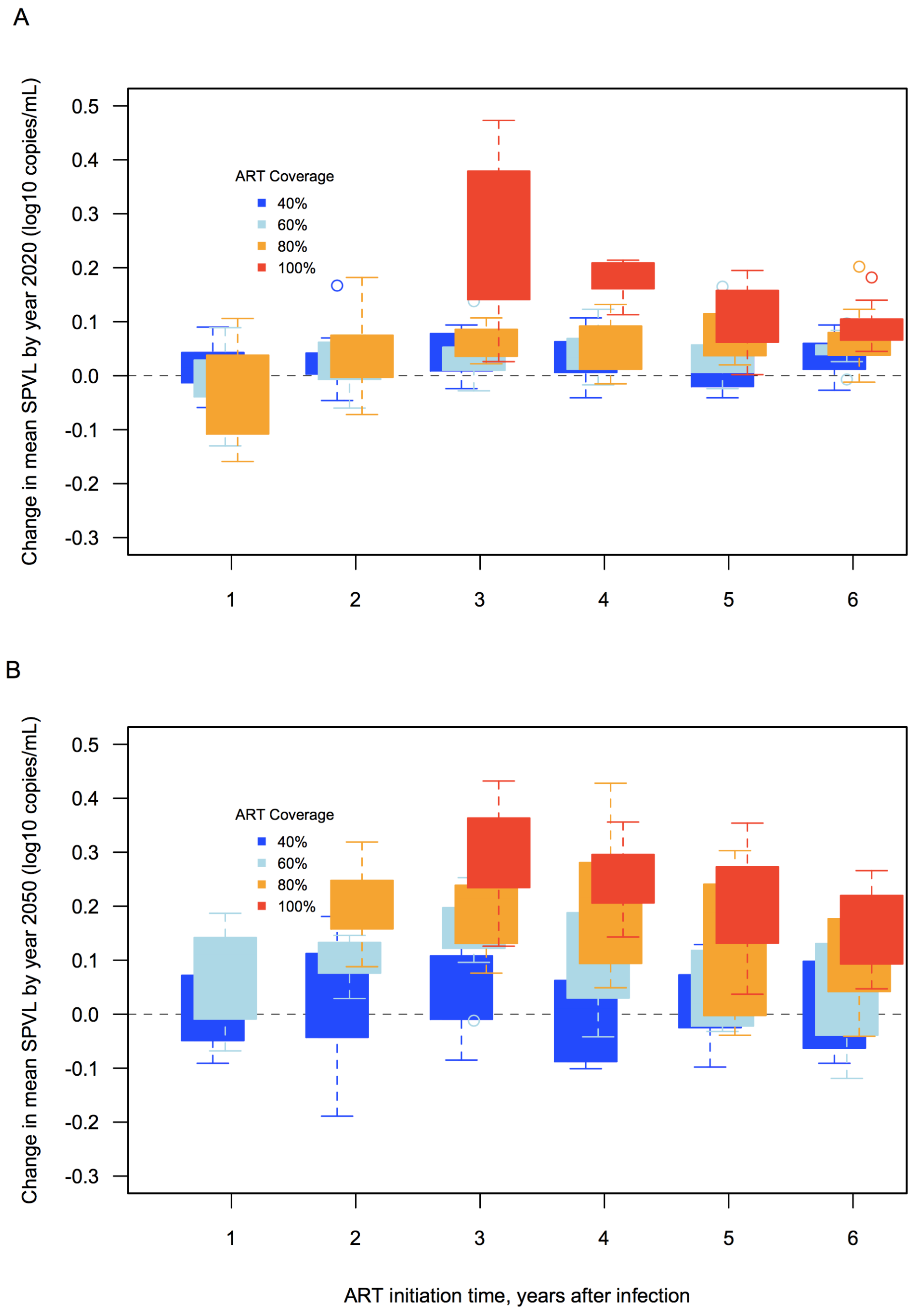

Supplementary Material Figure S3. Simulated effects of ART on HIV incidence, for scenarios when ART is initiated based on time since infection, at increasing coverage levels, measured at **A**) Eight and **B)** 38 years after rollout of ART (from 2012 to 2020 and 2050). ART initiation is determined based on time since infection.

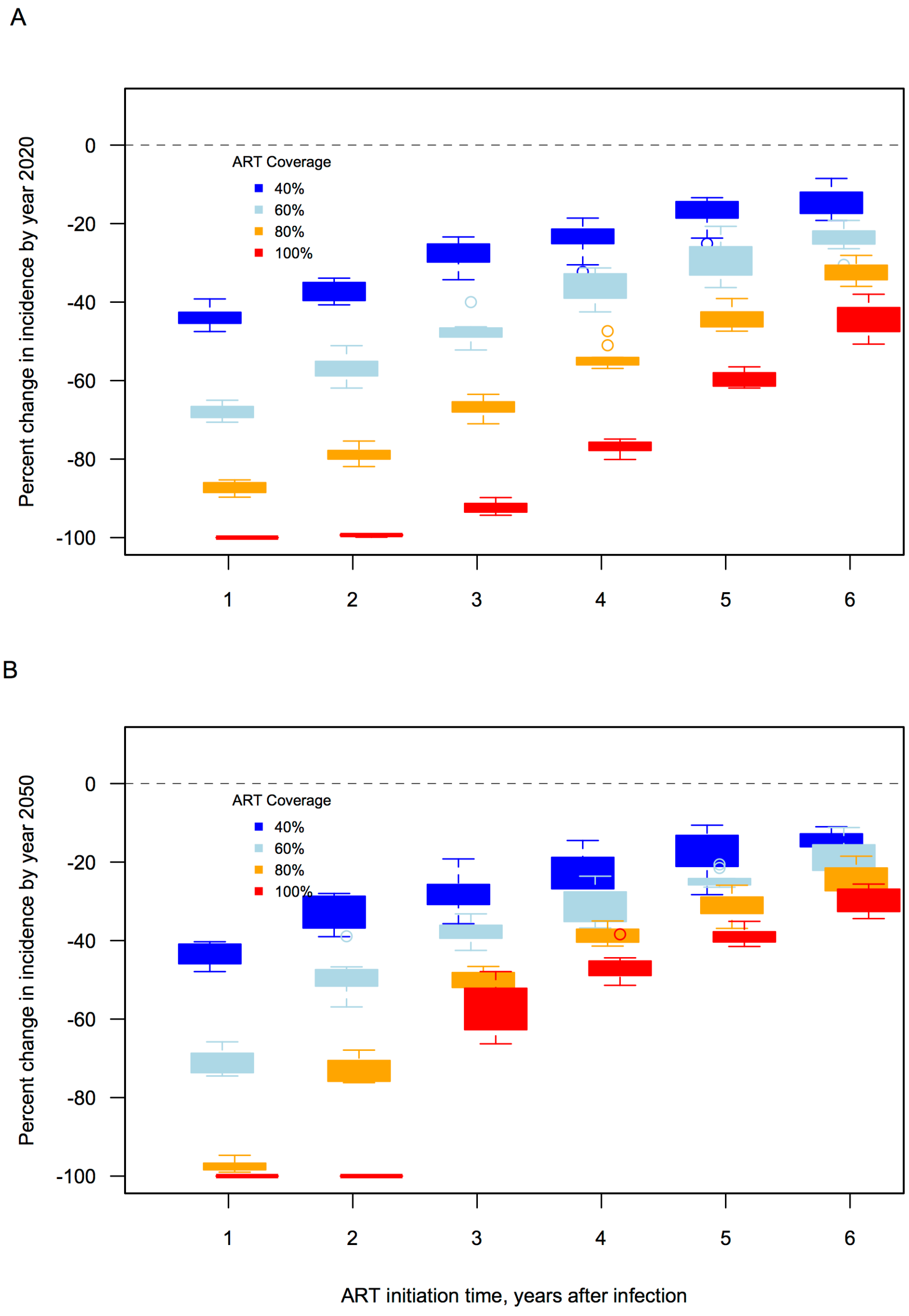

Supplementary Material Figure S4. Simulated effects of ART on HIV virulence evolution, for scenarios when ART is initiated based on time since infection, at increasing coverage levels. Shown are LOESS regression lines for ten random replicates for each ART coverage scenario (thin lines), and the mean of these replicates (thick lines). Initial mean SPVL was 4.5 log_{10} copies/mL. ART coverage lines start at year 22, corresponding to a simulation starting at year 1990 with ART rollout at year 2012. Truncated lines are the result of epidemic runs ending with 0% incidence.

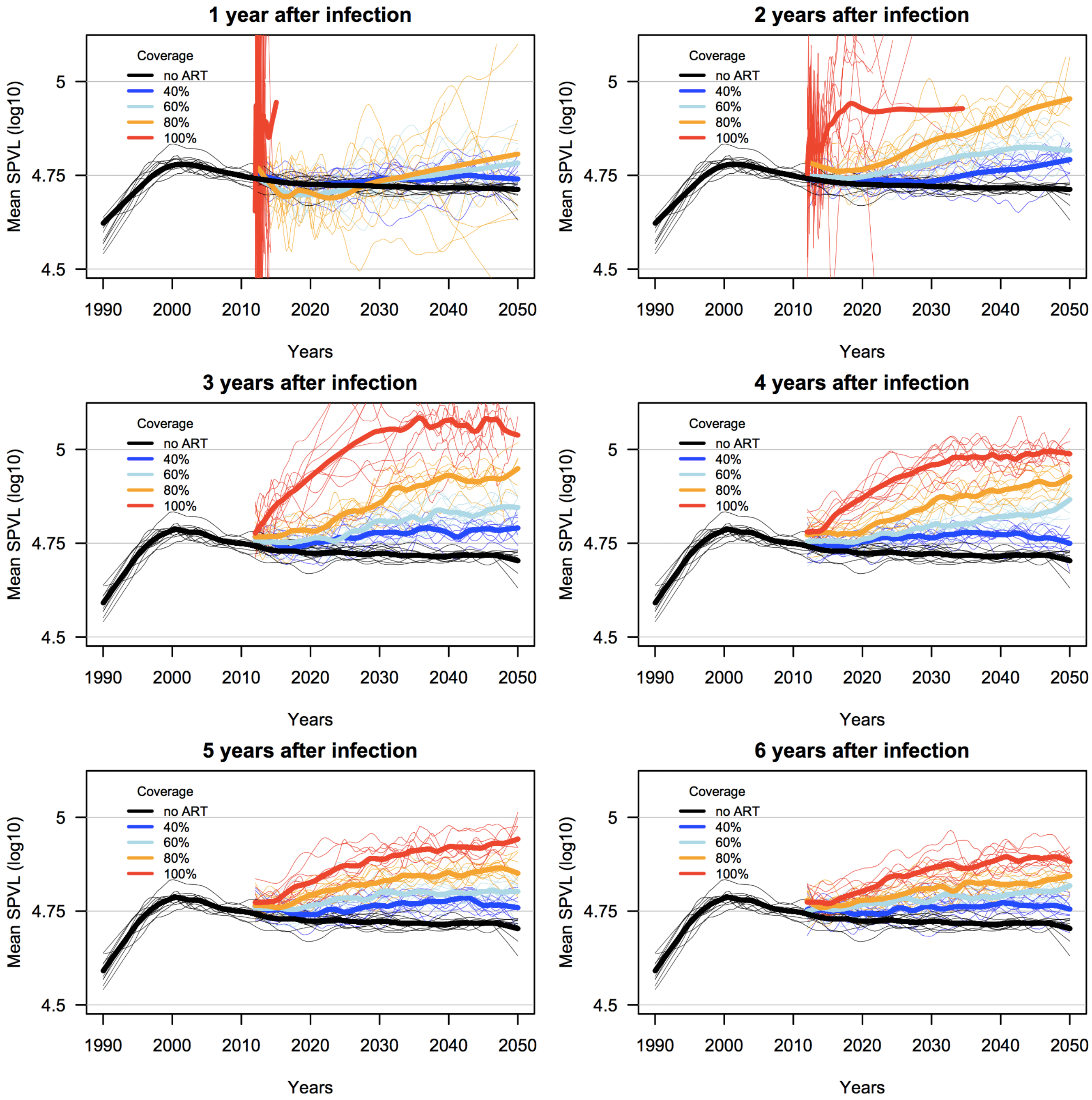

Supplementary Material Figure S5. Simulated effects of ART on HIV virulence evolution, measured at **A)** 8 and **B)** 38 years after rollout of ART (from ART rollout in 2012 to 2020 and 2050). ART initiation is determined based on CD4 count eligibility thresholds (shown on the X-axis). (Increases in mean SPVL for 100% coverage are not reported for CD4>500 because incidence is 0% in those scenarios.)

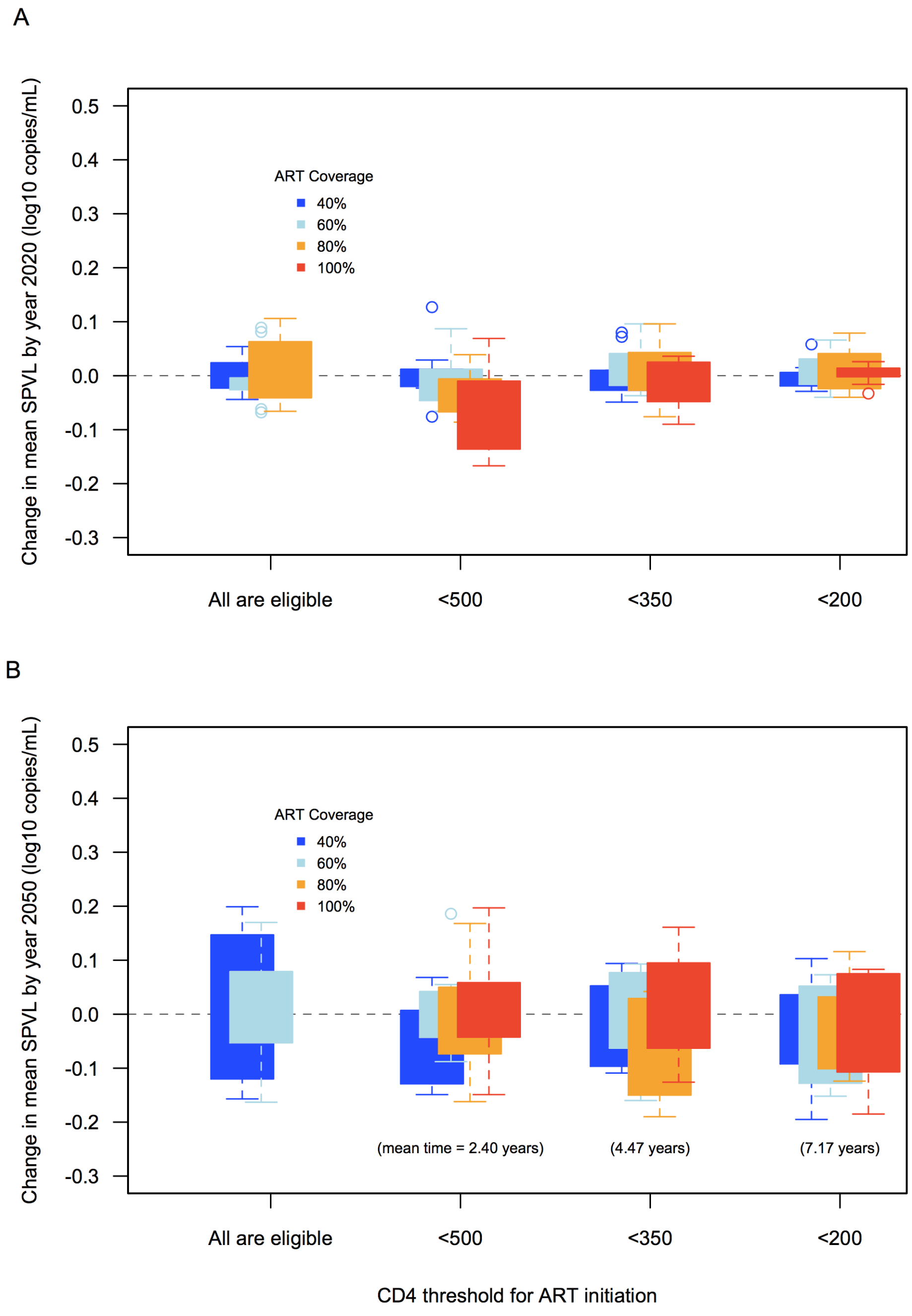

Supplementary Material Figure S6. Effects of ART on HIV incidence, measured at A) eight and B) 38 years after rollout of ART (from 2012 to 2020 and 2050). ART initiation is determined based on CD4 count eligibility thresholds.

A

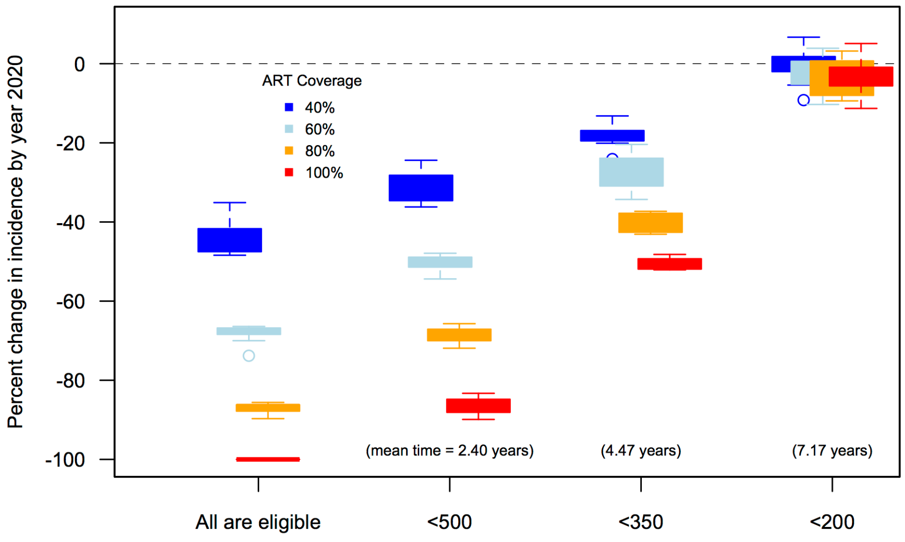

B

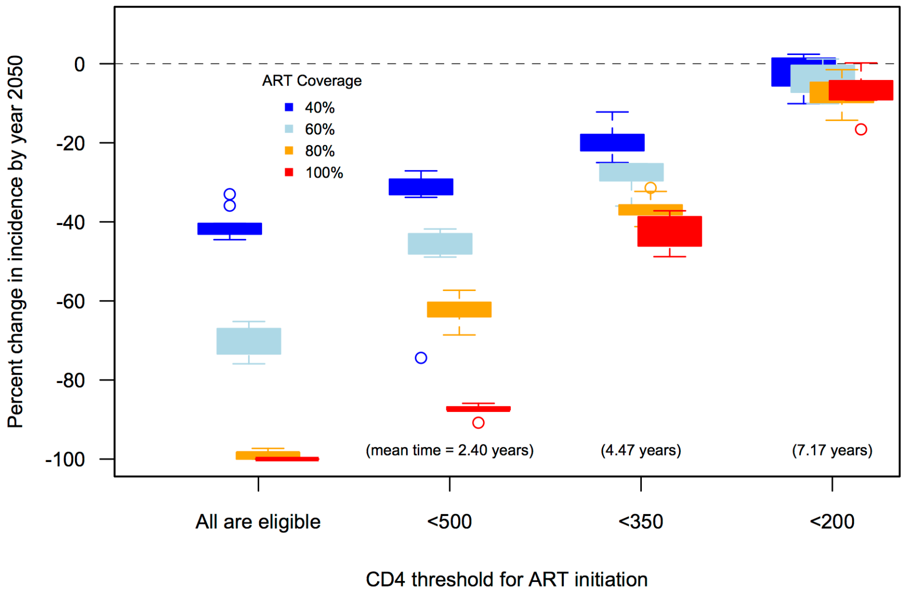

Supplementary Material Figure S7. Simulated effects of ART on HIV virulence evolution, for scenarios when ART is initiated based on CD4+ T cell count, at increasing coverage levels. Shown are LOESS regression lines for ten random replicates for each ART coverage scenario (thin lines), and the mean of these replicates (thick lines). Initial mean SPVL was 4.5 log_{10} copies/mL. ART coverage lines start at year 22, corresponding to a simulation starting at year 1990 with ART rollout at year 2012. Truncated lines are the result of epidemic runs ending with 0 incidence.

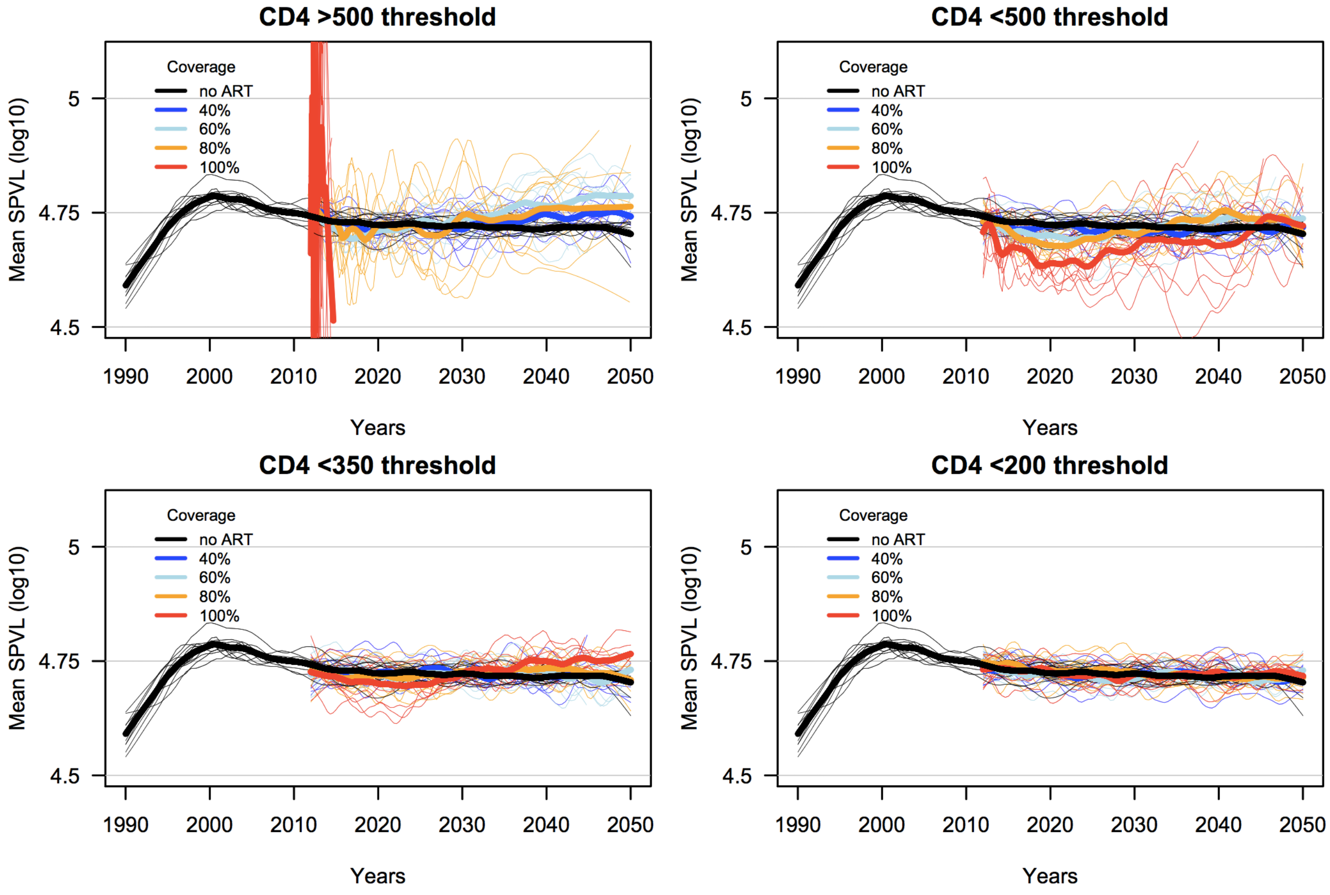

Supplementary Figure S8. Alternate model results: simulated trends in HIV incidence for ART scenarios of 40, 60, 80 and 100% coverage (individual probability of receiving ART with complete adherence) and CD4 count threshold for treatment initiation <350 cells/mL, versus the counterfactual epidemic simulation with no ART. Shown are LOESS regression lines for ten random replicates for each ART coverage scenario (thin lines), and the mean of these replicates (thick lines). Initial mean SPVL was 4.5 $\log_{10}$ copies/mL.

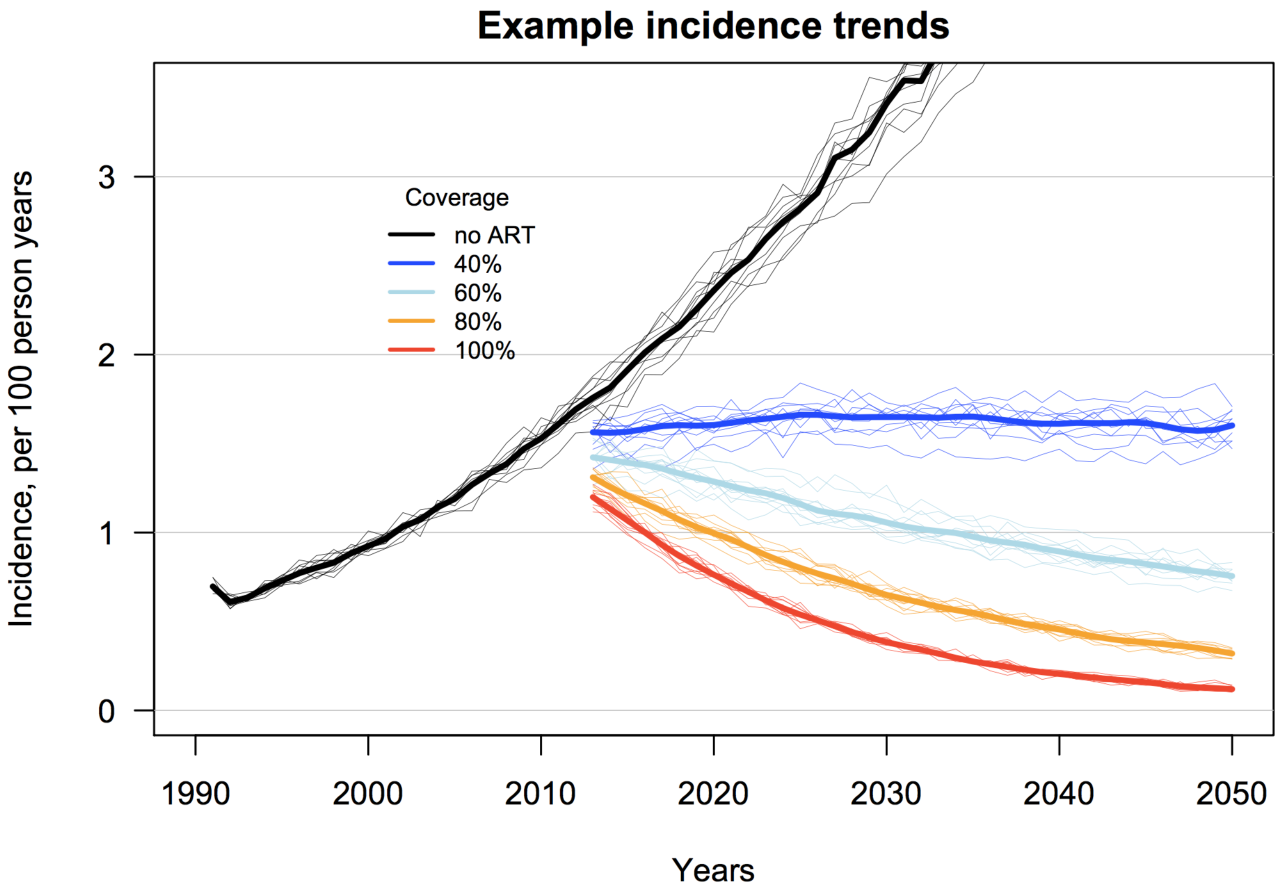

Supplementary Material Figure S9. Alternate (different calibration) model results: surface plots showing change in mean SPVL between epidemic simulations with and without ART, for scenarios of increasing ART coverage (individual treatment probability) and ART initiation based either on time since infection or CD4 count threshold. For epidemic scenarios with ART initiation based on time since infection, **A)** shows mean SPVL change 8 years after ART rollout (from year 2012 to year 2020), and **B)** shows mean SPVL change 38 years after rollout (from year 2012 to year 2050). For epidemic scenarios with ART initiation based on CD4 count, **C)** and **D)** show mean SPVL at 8 and 38 years after rollout, respectively.

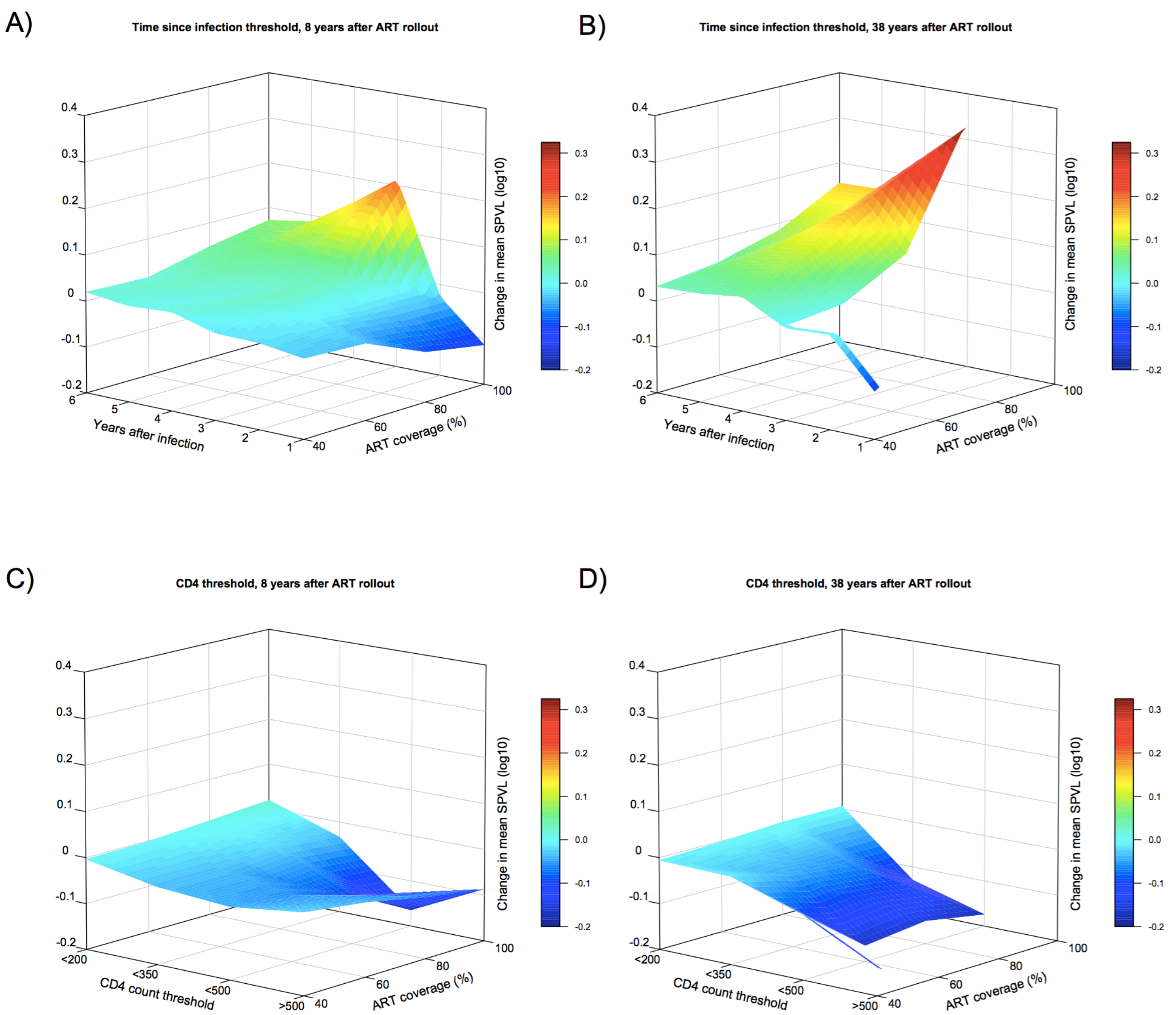

Supplementary Material Figure S10. Alternate model results: simulated effects of ART on HIV virulence evolution, for scenarios when ART is initiated based on time since infection, at increasing coverage levels. Shown are LOESS regression lines for ten random replicates for each ART coverage scenario (thin lines), and the mean of these replicates (thick lines). Initial mean SPVL was 4.5 $\log_{10}$ copies/mL. ART coverage lines start at year 22, corresponding to a simulation starting at year 1990 with ART rollout at year 2012. Truncated lines are the result of epidemic runs ending with 0 incidence.

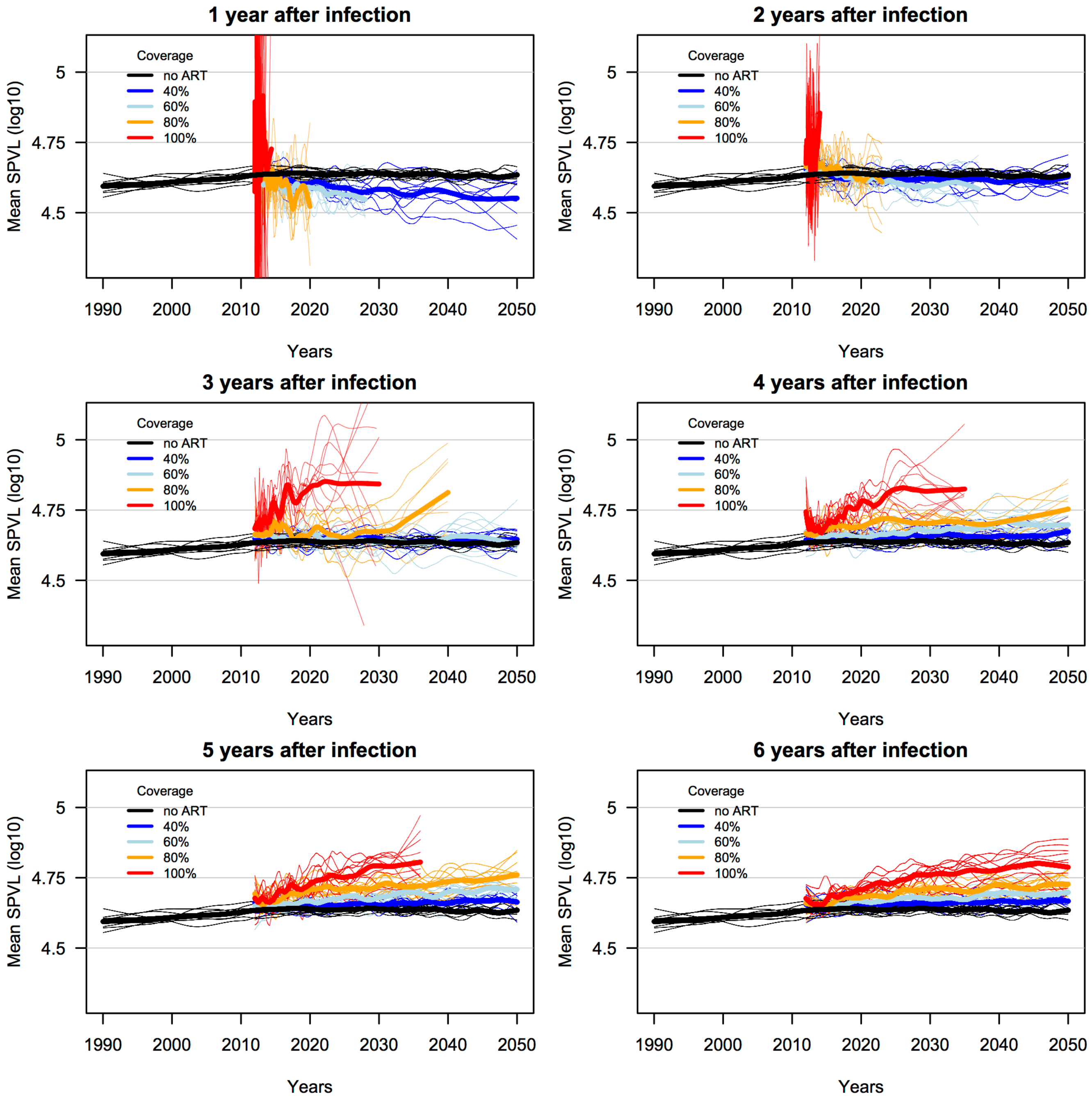

Supplementary Material Figure S11. Alternate model results: simulated effects of ART on HIV virulence evolution, for scenarios when ART is initiated based on CD4+ T cell count, at increasing coverage levels. Shown are LOESS regression lines for ten random replicates for each ART coverage scenario (thin lines), and the mean of these replicates (thick lines). Initial mean SPVL was 4.5 $\log_{10}$ copies/mL. ART coverage lines start at year 22, corresponding to a simulation starting at year 1990 with ART rollout at year 2012. Truncated lines are the result of epidemic runs ending with 0 incidence.

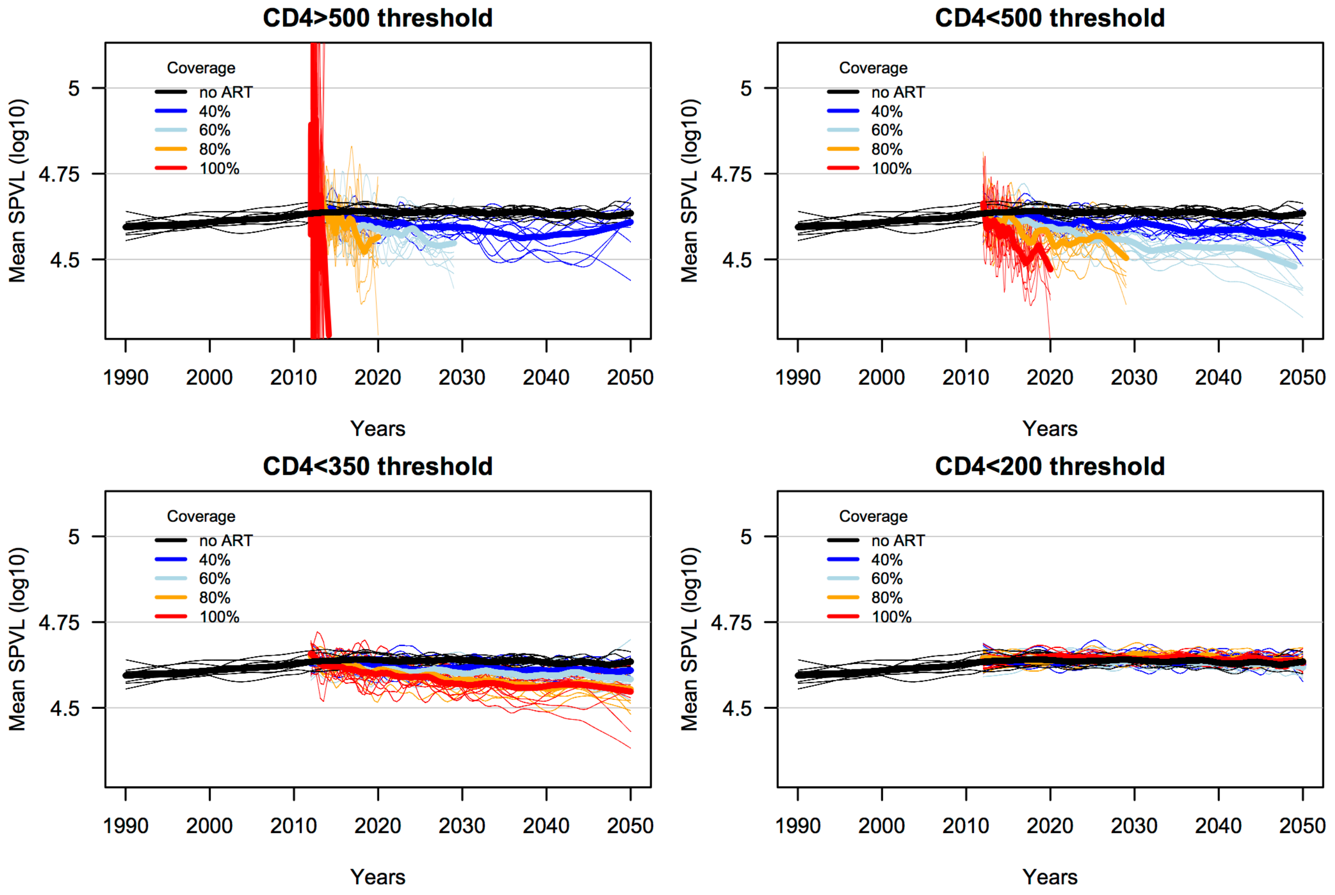

Supplementary Material Figure S12. Proportion of transmissions from SPVL lineages with higher (>4.74 log_{10} copies/mL) and lower (<4.74 log_{10}) levels than the population mean prior to ART rollout, for a scenario of 80% ART coverage and two different ART initiation scenarios: A) ART eligibility based on CD4<350; or B) ART eligibility at four years elapsed after infection.

A

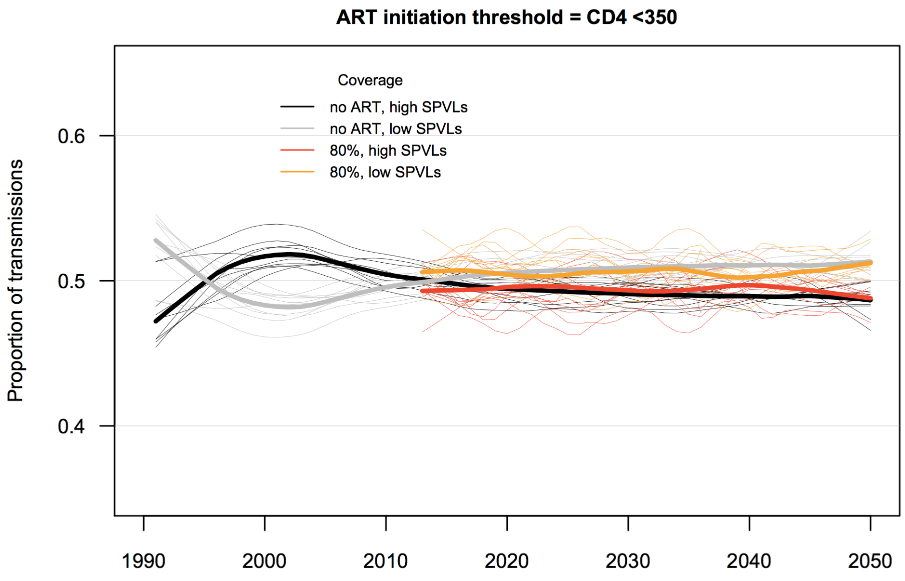

B

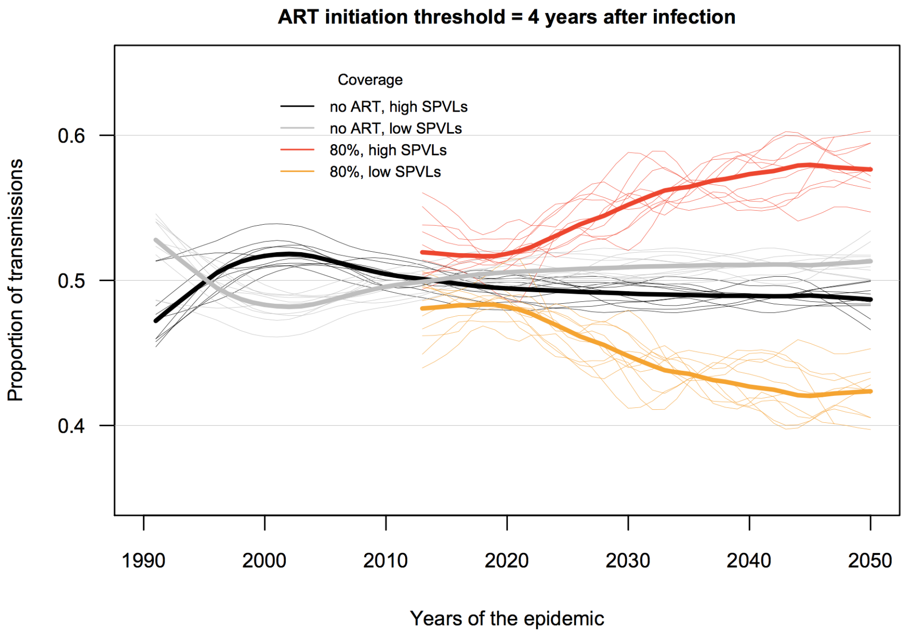

Supplement: Supplementary Data [file vew028supplementary.pdf]
